# Radioprotective effects of roxadustat (FG‐4592) in haematopoietic system

**DOI:** 10.1111/jcmm.13937

**Published:** 2018-10-18

**Authors:** Pei Zhang, Jicong Du, Hainan Zhao, Ying Cheng, Suhe Dong, Yanyong Yang, Bailong Li, Fu Gao, Xuejun Sun, Jianming Cai, Cong Liu

**Affiliations:** ^1^ Department of Radiation Medicine Faculty of Naval Medicine Second Military Medical University Shanghai China; ^2^ Department of Radiation Oncology Chinese PLA General Hospital Beijing China; ^3^ Department of Navy Aviation Medicine Faculty of Naval Medicine Second Military Medical University Shanghai China

**Keywords:** FG‐4592, HIF, irradiation, radioprotection

## Abstract

**Background:**

Ionizing radiation often causes severe injuries to radiosensitive tissues, especially haematopoietic system. Novel radioprotective drugs with low toxicity and high effectiveness are required. Prolyl hydroxylases domain (PHD) inhibitors have been reported to protect against radiation‐induced gastrointestinal toxicity. In this study, we demonstrated the protective effects of a PHD inhibitor, roxadustat (FG‐4592), against radiation‐induced haematopoietic injuries in vitro and in vivo.

**Methods:**

Tissue injuries were evaluated by Haematoxilin‐Eosin (HE) staining assay. HSCs were determined by flow cytometry with the Lin^−^Sca‐1^+^c‐Kit^+^ (LSK) phenotype. Cell apoptosis was determined by Annexin V/PI staining assay. Immunofluorescence was performed to measure radiation‐induced DNA damage. A western blot assay was used to detect the changes of proteins related to apoptosis.

**Results:**

We found that FG‐4592 pretreatment increased survival rate of irradiated mice and protected bone marrow and spleen from damages. Number of bone marrow cells (BMCs) and LSK cells were also increased both in irradiated mice and recipients after bone marrow transplantation (BMT). FG‐4592 also protected cells against radiation‐induced apoptosis and double strand break of DNA.

**Conclusions:**

Our data showed that FG‐4592 exhibited radioprotective properties in haematopoietic system both in vivo and in vitro through up‐regulating HIF‐1α, indicating a potential role of FG‐4592 as a novel radioprotector.

## 1 INTRODUCTION

Exposure to ionizing radiation often leads to severe damages, which is a serious military and public health concern. Acute radiation syndrome occurs after total body exposure to radiation at doses more than 1 Gy.^1^, ^2^, ^3^ Supportive care and bone marrow transplant could be effective treatments to haematopoietic injuries.^2^ However, exposure to higher dose (more than 10 Gy) of radiation usually results to intestinal injuries, which, unfortunately, lack effective treatments and often lead to death.^4^ At present, thiol compounds are the most effective radioprotectors and the only FDA approved drug is WR2721, which shows much toxicity.^5^, ^6^, ^7^ As a result, it is urgently required to find more effective and safe radioprotective compounds.

Hypoxia‐inducible factors (HIFs) are transcription factors that regulate expressions of genes related to reduced oxygen (hypoxia).^8^, ^9^ The stability of HIFs is regulated by prolyl hydroxylases domain (PHD)‐containing proteins which hydroxylate the HIF‐α subunit and lead to its proteasomal degradation.^10^ Recently, studies have revealed that PHD inhibitors showed strong radioprotective properties. Culllen et al reported PHD inhibitor (DMOG) could protect against radiation‐induced gastrointestinal toxicity.^11^ It is also experimentally established that pharmacologic stabilization of HIF‐1α increased haematopoietic stem cell quiescence in vivo and accelerated blood recovery after severe irradiation.^12^ Therefore, it is strongly suggested that PHD could exhibit radioprotective properties in haematopoietic system. Roxadustat, also known as FG‐4592, is an oral PHD inhibitor and is used for the treatment of anaemia in patients with chronic kidney disease (CKD).^13^, ^14^ We hypothesized whether FG‐4592 could alleviate radiation‐induced injuries as a HIF regulator.

In present study, we demonstrated FG‐4592 protected mice and cultures cells from radiation‐induced injuries, indicating its strong radioprotective properties.

## 2 MATERIALS AND METHODS

### 2.1 Irradiation

^60^Co‐γ rays at Irradiation Center (Faculty of Naval Medicine, Second Military Medical University, China) was used for irradiation. Mice and cultured cells were exposed to different doses of radiation according to the requirement of the present study.

### 2.2 Mice and treatments

All experiments were approved by the Second Military Medical University, China in accordance with the Guide for Care and Use of Laboratory Animals published by the US NIH (Publication No. 96‐01). Male wild‐type C57 mice (6‐8 weeks old) were purchased from China Academy of Science (Shanghai, China). Mice were housed in individual cages in a temperature‐controlled room with a 12 hours light/dark cycle. Food and water were provided ad libitum. FG‐4592 (25 mg/kg) was daily delivered via the intraperitoneal administration for 7 days before irradiation. Mice were sacrificed by cervical dislocation at different time after irradiation.

### 2.3 Cell culture and treatment

AHH‐1 cells (American Type Culture Collection, Manassas, VA, USA) were maintained in 1640 (Gibco) medium with 10% fetal bovine serum (Gibco) and 1% penicillin‐streptomycin‐glutamine (Hyclone, Logan, UT, USA) at 37°C in a 5% CO_2_ humidified chamber. Primary bone marrow cells and splenocytes were procured at 12 hours before irradiation from C57 mice and cultured in the same condition. After FG‐4592(Medchemexpress, MCE, USA) treatment (20 μ mol L^−1^), cells were exposed to different doses of γ‐irradiation and used in next experiments.

### 2.4 Survival assays

Survival rates of the mice were recorded daily for 30 days after radiation with 7.5 Gy at a dose rate of 1.5 Gy/min.

### 2.5 Tissues isolation and Haematoxilin‐Eosin (HE) staining

On day 0, day 3, and day 7 post total body irradiation, murine spleen, and femur were isolated, fixed and subjected to sections. HE staining method was applied to detect tissue damages as previously described.^15^

### 2.6 Transplantation experiments

For transplantation of total bone marrow cells, adult C57BL/6 (8 weeks of age) were irradiated with a dose of 7.5 Gy total body irradiation with a dose rate of 1.5 Gy/min. These mice were then reconstituted with 2.5 × 10^6^ freshly isolated bone marrow cells from mice treated with or without FG‐4592 in 0.2 mL PBS within 24 hours of irradiation.

### 2.7 Immunofluorescence analysis

Immunofluorescence analysis was performed to detect γ‐H2AX foci in order to evaluate the DNA damage. Briefly, after irradiation AHH‐1 cells in different groups were fixed in 4% paraformaldehyde for 20 minutes and permeabilized in 0.5% Triton X‐100 for 10 minutes. After blocked in serum, cells were stained with γ‐H2AX primary antibody and then secondary antibody. Then, cells were stained with DAPI for 5 minutes. Cellular images were obtained using Olympus BX60 fluorescent microscope (Olympus America Inc., Center Valley, PA, USA) with a Retiga 2000R digital camera (Q Imaging Inc., Surrey, BC, Canada).

### 2.8 Flow cytometry analysis

Before analysis of samples (bone marrow cells and splenocytes), erythrocytes were first removed by lysis buffer and cells were stained with various antibodies. For analysis of HSCs (LSK cells), we used antibodies as follows: Lineage cell detection cocktail‐Biotin (Miltenyi Biotec), Streptavidin‐FITC (eBioscience), Sca‐1‐PC5.5 (eBioscience), and c‐Kit‐APC (eBioscience).

At different times after irradiation at a dose of 6 Gy, apoptosis of AHH‐1, bone marrow cells and splenocytes were determined by double‐staining with Annexin V‐FITC and Propidium Iodide (PI) using Apoptosis Detection Kit (Invitrogen, Carlsbad, California, USA) and analyzed by flow cytometry (Beckman Cytoflex) according to the manufacturer instructions.

### 2.9 Antibodies and Western blotting analysis

At different times after treatment, the proteins from AHH‐1 were obtained using M‐PER Mammalian Protein Extraction Reagent (Thermo, USA) according to manufacturer's protocol, and then analyzed by Western blotting to detect Bcl‐2 (Cell Signaling Tech, 1:1000), Bax (Cell Signaling Tech, 1:1000), cleaved‐caspase3 (Proteintech, Wuhan, China, 1:1000), cyt‐c (Cell Signaling Tech, 1:1000), and β‐actin (Proteintech, Wuhan, China, 1:1000). The secondary antibody (1:5000) was also purchased from Cell Signaling Tech.

### 2.10 Statistical analysis

Data are shown as means ± the standard error of mean (SEM) for each experiment. Statistical analysis was performed using One Way Analysis of Variance. Between groups, variance was determined using the Student‐Newman‐Keuls post hoc test. A *P*‐value of less than 0.05 was considered to be statistically significant.

## 3 RESULTS

### 3.1 FG‐4592 increased survival rates of irradiated mice

**Figure 1:**
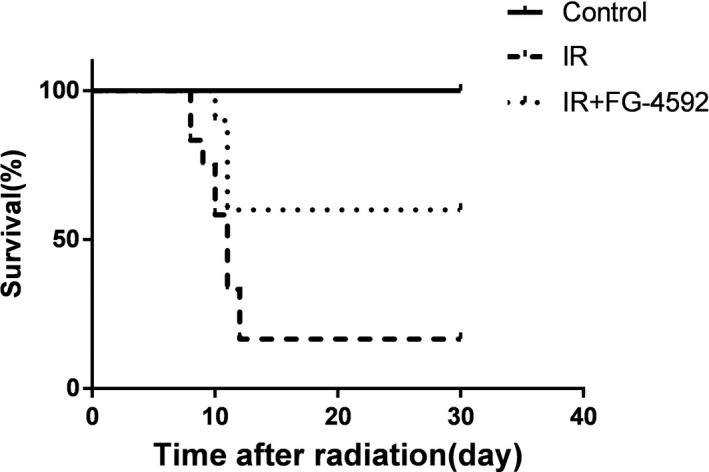
FG‐4592 increases the survival rates of irradiated mice. Mice irradiated at a dose of 7.5 Gy were pretreated with or without FG‐4592. The survival rate of irradiated mice is 16.7%, while FG‐4592 pretreatment increases the survival rate to 60%

Eighty percent of irradiated mice without FG‐4592 treatment died by the 12th day after radiation at a dose of 7.5 Gy, while 60% of mice pretreated with FG‐4592 (25 mg/kg) survived (Figure 1). Thus, FG‐4592 could protect mice from radiation‐induced injury.

### 3.2 FG‐4592 protected bone marrow and spleen against radiation

**Figure 2:**
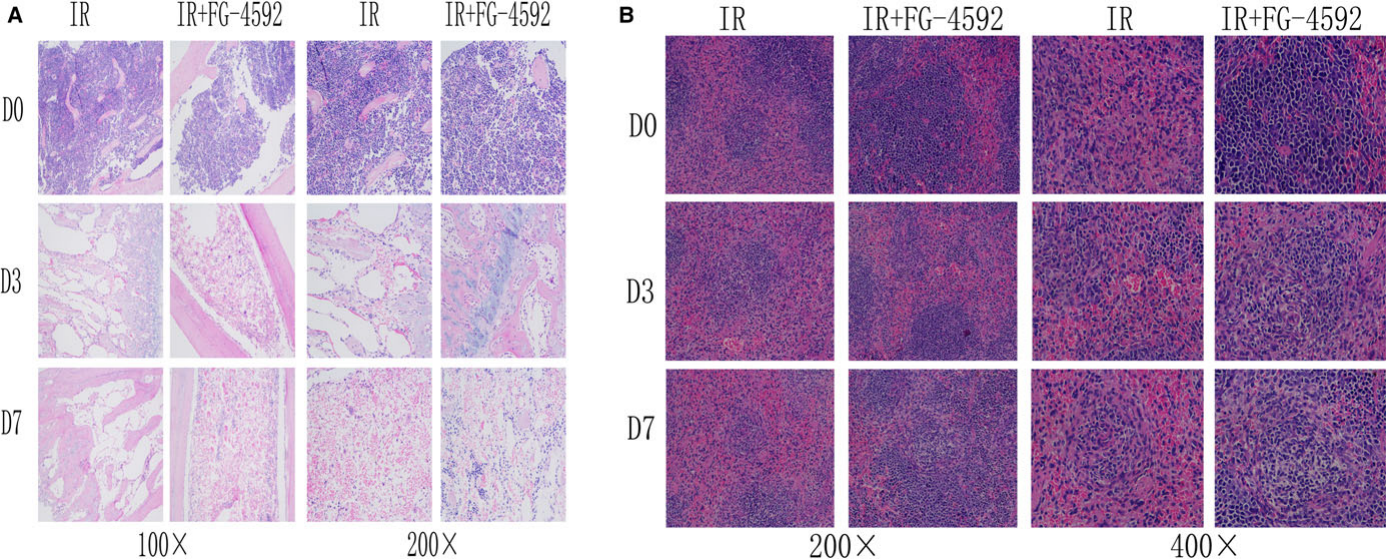
FG‐4592 protects bone marrow and spleen against radiation. Mice were pretreated with FG‐4592 daily for a week before irradiation at a dose of 7.5 Gy. On day 3 and day 7 after irradiation, femurs, and spleen were isolated and subjected to HE staining. Results show that FG‐4592 alleviates radiation‐induced injuries in bone marrow (A) and spleen (B)

Bone marrow is sensitive to ionizing radiation. Radiation‐induced damages caused reduced nucleated cells and structural destruction in bone marrow. Murine femurs were isolated and histological sections were performed to evaluate the damages of bone marrow. In FG‐4592 treated group, radiation‐induced injuries on bone marrow were significantly alleviated and nucleated cells were protected (Figure 2A). Playing important roles in regard to immune system, spleen is also quite sensitive to radiation. To determine whether FG‐4592 could alleviate radiation‐induced injuries on spleen, the structure of spleen was analyzed. We found that irradiated spleen exhibited reduced size of white pulps after radiation. However, in FG‐4592 treated group, the destructive damages were mitigated (Figure 2B).

### 3.3 Radioprotective effects of FG‐4592 on HSCs in vivo

**Figure 3:**
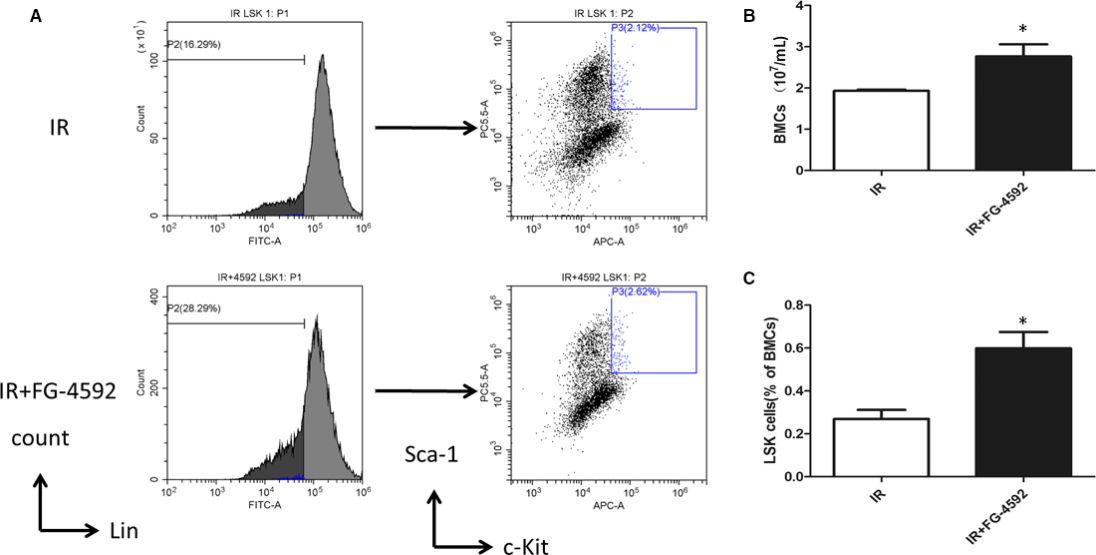
Radioprotective effects of FG‐4592 on HSCs in vivo. Mice were irradiated at a dose of 7.5 Gy after pretreatment with FG‐4592. LSK(Lin^−^ Sca‐1^+^ c‐Kit^+^) cells were determined by flow cytometry (A). FG‐4592 treatment exhibits a larger pool of HSCs (C) and increases number of bone marrow cells (B)

Hematopoiesis depends on the unique ability of few haematopoietic stem cells (HSCs)to self‐renew and to generate differentiated progeny of all blood cell lineages. To determine whether FG‐4592 confers a protective advantage of HSCs to irradiation in vivo, we exposed mice treated with or without FG‐4592 to radiation (7.5 Gy) and analyzed the levels of LSK cells in the bone marrow. As is shown in Figure 3, LSK cells increased significantly in FG‐4592 treated bone marrow 24 hours after radiation. As well, FG‐4592 treatment exhibited increased BMC numbers after radiation.

### 3.4 FG‐4592 enhanced hematopoiesis after bone marrow transplantation

**Figure 4:**
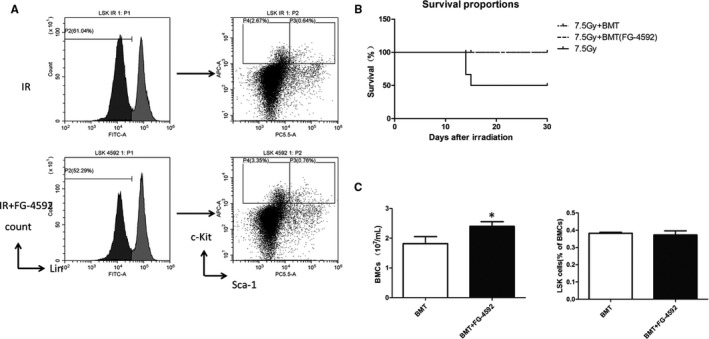
FG‐4592 enhances hematopoiesis after bone marrow transplantation. We performed bone marrow transplantation(BMT) to validate whether FG‐4592 treatment could improve HSC function. Survival analysis shows that Aal recipients survived. In contrast, approximately half of the irradiated mice die within 2 wk (B). Further, recipients with FG‐4592 treatment exhibits increased number of bone marrow cells and LSK cells (A and C)

Our results showed that FG‐4592 protected bone marrow from radiation‐induced injury and increased HSC population after radiation. Furthermore, we performed bone marrow transplantation (BMT) to validate whether FG‐4592 treatment could improve HSC function. BMCs isolated from mice treated with or without FG‐4592 were transplanted to recipients in 24 hours after radiation at a dose of 7.5 Gy. All recipients survived. In contrast, approximately half of the irradiated mice died within 2 weeks (Figure 4B). We examined the haematopoietic system of recipients and found that recipients with FG‐4592 treatment exhibited an increased number of WBC in peripheral blood (Table 1). However, the number of RBC showed a statistically insignificant increase in recipients treated with FG‐4592 (Table 1). In addition, reconstituted recipient mice with FG‐4592 treatment showed increased BMC number and spleen weight. Moreover, recipients with a treatment of FG‐4592 had a larger pool of HSCs. All these suggested that FG‐4592 improved hematopoiesis.

**Table 1:** Hematopoietic parameters

Parameter	Recipients	Recipients plus FG‐4592 treatment
Blood		
WBC (×10^3^/μL)^a^	5.2 ± 1.60	8.8 ± 1.95
RBC (×10^6^/μL)	9.67 ± 1.35	10.31 ± 0.69
Hemoglobin (g/dL)	13.9 ± 2.17	15.2 ± 1.14
Platelet (×10^3^/μL)	1080.7 ± 235	985.3 ± 69.5
Differential WBC (%)		
Lymphocytes	67.8 ± 5.04	68.7 ± 6.46
Monocytes	3.1 ± 0.29	4.1 ± 0.8S
Granulocytes	29.1 ± 5.00	27.1 ± 6.12
Tissues		
WBC/femur (×10^3^/μL)^a^	18.1 ± 3.95	23.8 ± 2.27
Spleen (mg)^a^	51.0 ± 3.99	86.7 ± 10.39
Spleen coefficient^a^	0.29 ± 0.015	0.39 ± 0.038
Thymus (×10^3^/μL)	28.1 ± 6.73	25.8 ± 3.41

White blood cells (WBC), red blood cells (RBC), haemoglobin. Haematocrit and platelets in transplanted recipient mice treated with or without FG‐4592 30 d after irradiation. Results are mean ± SEM.

a *P *<* *0.05.

### 3.5 FG‐4592 protected cells against radiation‐induced apoptosis

**Figure 5:**
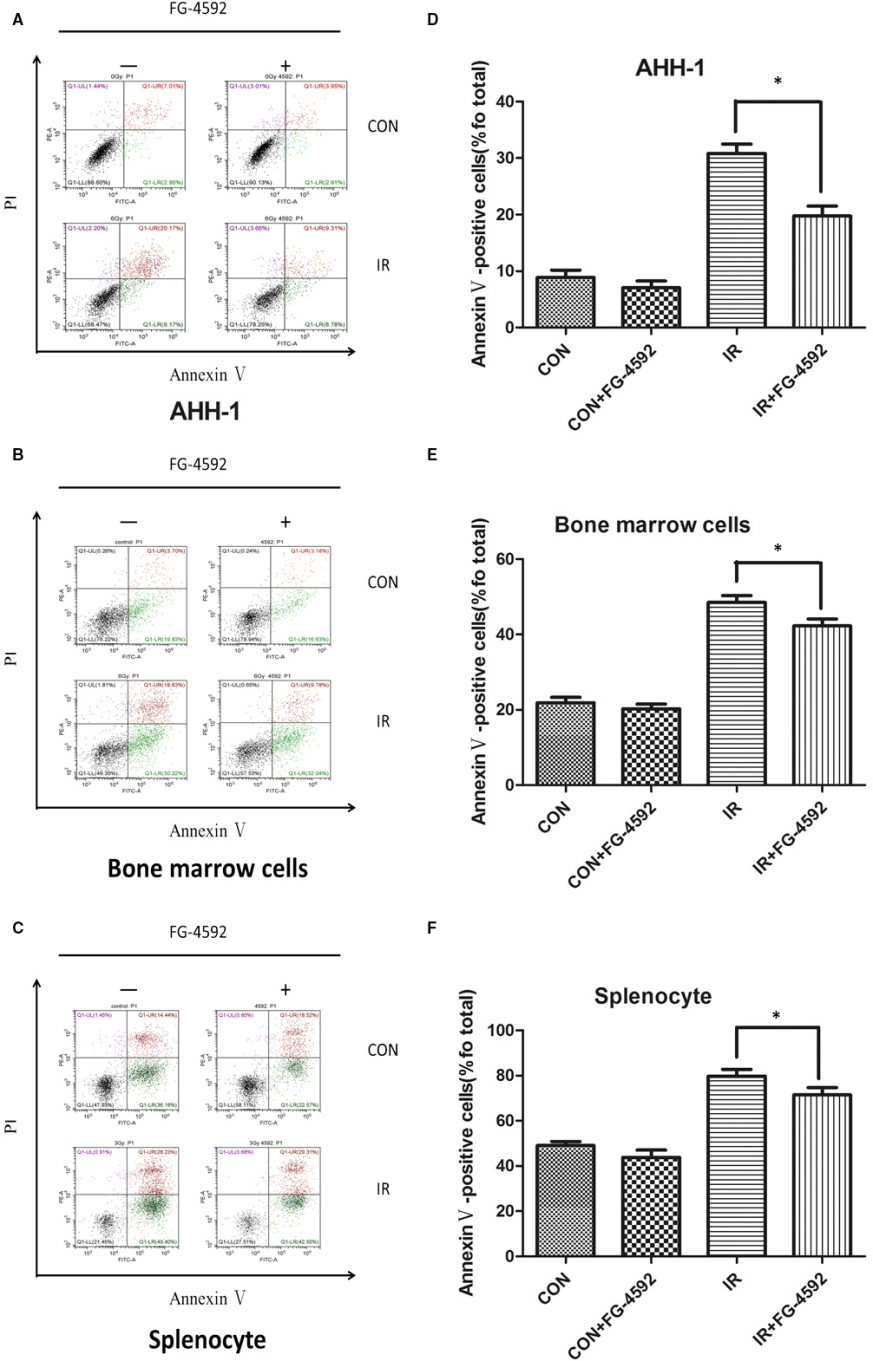
FG‐4592 protects cells from radiation‐induced cell death. AHH‐1, bone marrow cells and splenocytes were pretreated with FG‐4592 (20 μ mol L^−1^) and then irradiated at a dose of 6 Gy. 24 h after irradiation, cell apoptosis was measured by double‐staining with Annexin V‐FITC and PI (A, B and C). Annexin V positive cells are qualified in different groups(D, E, and F). (n = 3),*P < 0.05 vs IR group

To evaluate whether FG‐4592 has radioprotective property in vitro, cell apoptosis was determined. In this study, AHH‐1, bone marrow cells and splenocytes were used. At 12 or 24 hours after irradiation, cell apoptosis was determined using flow cytometry. Our results showed that cell apoptosis increased obviously after irradiation. However, in FG‐4592 treatment group, cell apoptosis was significantly inhibited, indicating a radioprotective role of FG‐4592 in vitro (Figure 5).

### 3.6 FG‐4592 protected cells from radiation‐induced DNA damage

**Figure 6:**
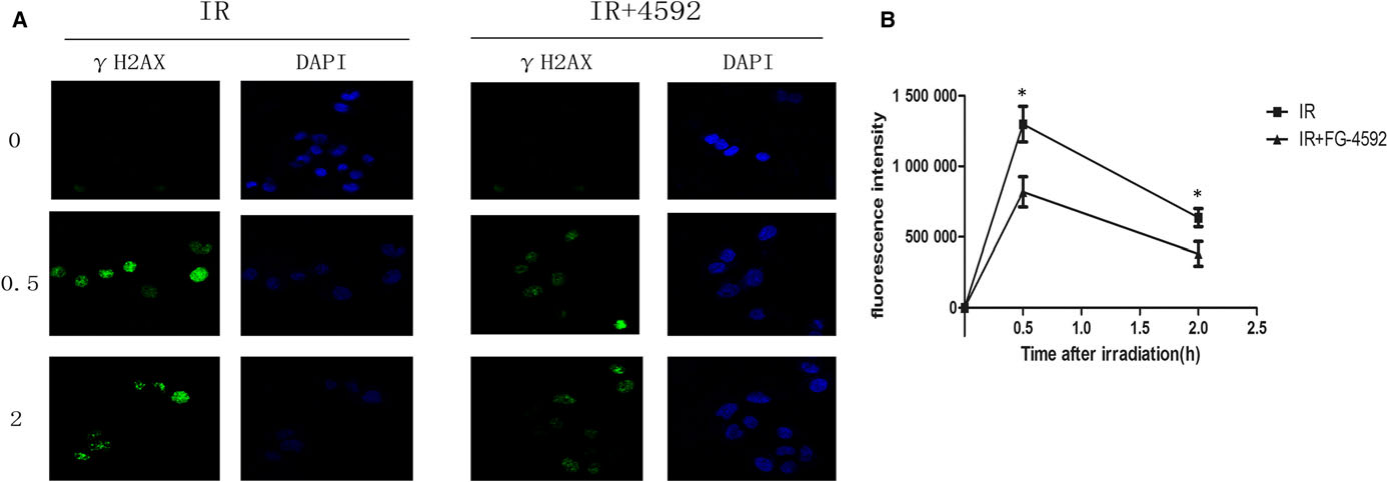
FG‐4592 mitigated radiation‐induced DNA injuries. AHH‐1 cells pretreated with or without FG‐4592 were exposed to 6 Gy irradiation and γ‐H2AX assay was used to determine DNA damages at 0, 0.5, 2 h after irradiation

As is well known that DNA is a main target of irradiation and severe DNA damage often leads to cell death. To evaluate the effect of FG‐4592 on DNA damage repair, γ‐H2AX foci assay was determined. Our results showed that FG‐4592 reduced the number of γ‐H2AX foci per cell at 0.5 and 2 hours after irradiation, indicating a role of promoting DNA damage repair (Figure 6).

### 3.7 FG‐4592 activated HIF1‐α and regulated apoptosis‐related molecules

**Figure 7:**
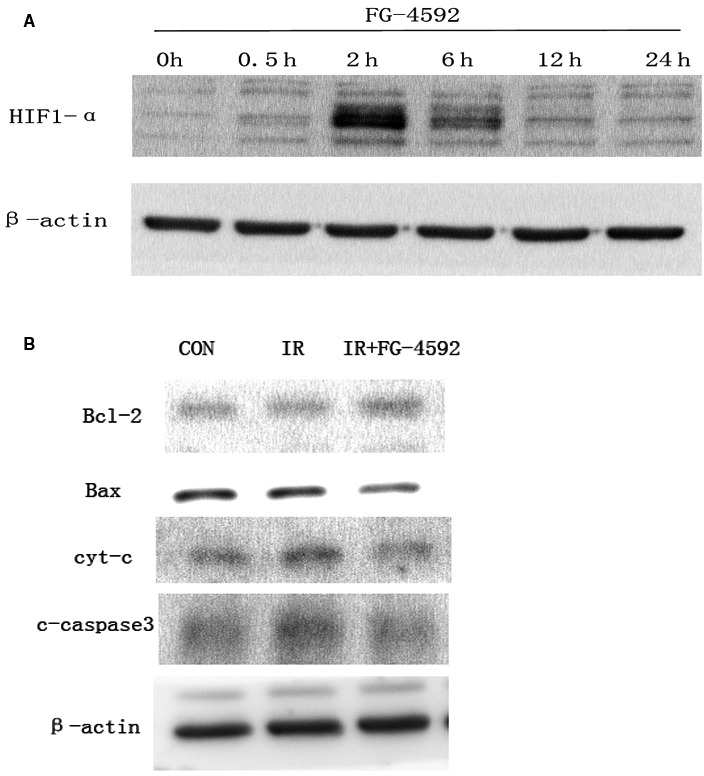
FG‐4592 increases level of HIF‐1α and regulates apoptosis‐related proteins in AHH‐1. The effects of FG‐4592 on HIF1‐α were detected using Western Blot. Level of HIF‐1α was measured at different time points after the treatment of FG‐4592 (A). Cell lysate was obtained at 6 h after irradiation and used to evaluate the regulation of FG‐4592 on apoptosis‐related molecules. Apoptosis promoting molecules (c‐caspase3 and cyt‐c) are down‐regulated and molecules that inhibit apoptosis are up‐regulated by FG‐4592 after irradiation in AHH‐1 (B)

The effect of FG‐4592 on HIF‐1α was determined. The level of HIF‐1α was measured after FG‐4592 treatment at different time points. Results showed that the level of HIF‐1α increased obviously at 2 h after FG‐4592 treatment (Figure 7A). During the progress of radiation‐induced apoptosis, many related molecules changed. In our study, the levels of apoptosis‐related molecules were measured. It showed that FG‐4592 treatment reduced apoptosis promoting proteins, which were up‐regulated by irradiation and increased proteins which inhibited apoptosis and were down‐regulated after irradiation (Figure 7B).

## 4 DISCUSSION

In our present experiment, we demonstrated that FG‐4592 showed strong radioprotective effects in cultured cells and mice. Our data showed that FG‐4592 increased the survival rate of mice irradiated at a dose of 7.5 Gy. Irradiation induced severe injuries on bone marrow and spleen which were sensitive to ionizing radiation. However, in FG‐4592 treated group, radiation‐induced injuries were significantly alleviated. Furthermore, FG‐4592 treatment increased the number of HSCs both in irradiated mice and recipients. We also found that FG‐4592 exerted radioprotective properties in vitro. It was showed that FG‐4592 protected cells from radiation‐induced apoptosis and promote DNA damage repair.

It is well known that ionizing radiation often causes severe injuries to radiosensitive tissues, which affects human health and limits the use of radiotherapy in patients.^16^ Unfortunately, few safe and effective drugs exist for the treatment of radiation‐induced injury. Recently, it was reported that PHD inhibition mitigated and protected against radiation‐induced gastrointestinal toxicity.^17^, ^18^ It was demonstrated that PHD inhibitor (DMOG) could protect against supralethal doses of ionizing radiation to the abdomen or whole body through up‐regulating hypoxia‐inducible factor‐2 (HIF‐2).^11^ The radioprotection of PHD inhibition remain unclear, but likely result from improved epithelial integrity of the gastrointestinal tract.^11^ The increased integrity of the GI tract allows the gut to maintain proper fluid homeostasis and barrier functions.^11^, ^19^

The role of PHD/HIF axis in radioprotection highlights the potential of these inhibitors as radioprotectors or mitigators. Previous studies have shown that roxadustat, also known as FG‐4592, is a first‐in‐class small molecule oral PHD inhibitor, which is currently used for the treatment of anaemia in patients with chronic kidney disease(CKD) in phase III clinical trials.^20^ So we evaluated the effects of FG‐4592 in radioprotection, which may facilitate its use as a protector after large‐scale accidental radiation incidents or as part of radiotherapy treatment in the future.^17^

In our study, we evaluated the radioprotective property of FG‐4592 in vitro and in vivo. Firstly, we investigated the effects of FG‐4592 on bone marrow and spleen, which are sensitive to ionizing radiation. We found that FG‐4592 alleviated damages on bone marrow structure and restored the number of nucleated cells. Also, injuries of spleen were mitigated after FG‐4592 treatment.

The HSC has the ability to perpetuate itself as well as to differentiate into mature blood cells of all lineages. In the mouse, HSCs can be isolated by their expression of undetectable levels of lineage markers (such as B220, CD3, Mac‐1) and high levels of c‐Kit and Sca‐1, which are known as the LSK cells.^21^ If HSC are injured by irradiation, long‐term, or permanent damage to the haematopoietic system occurs and bone marrow failure and death of the organism may occur.^22^ In the study, we examined whether FG‐4592 could protect HSCs from irradiation‐induced injury. Our data showed that mice treated with FG‐4592 had a larger pool of HSCs after irradiation. In addition, we performed bone marrow transplantation and proved that FG‐4592 treatment enhanced hematopoiesis in recipients.

Radiation interacts with DNA and causes different types of DNA damages including double strand break or single strand break.^23^, ^24^ The number of γ‐H2AX foci per cell was used as a marker of DSB, and in this study we determined γ‐H2AX kinetics in AHH‐1 cells. Results showed FG‐4592 mitigated DNA damages which were induced by irradiation. Meanwhile, cell apoptosis in response to radiation was also inhibited with FG‐4592 treatment. These data suggested FG‐4592 could exert protective effect in vitro.

In conclusion, our data demonstrated that FG‐4592 effectively protected haematopoietic system from radiation‐induced injuries in vitro and in vivo. Also, as a PHD inhibitor with high safety and low toxicity which has already been in Phase III development, FG‐4592 showed promising potential to be a new radioprotectant.

## CONFLICTS OF INTEREST

The authors have no conflicts of interest to disclose.
